# Adverse Events Associated With the Use of Sipuleucel-T Reported to the US Food and Drug Administration’s Adverse Event Reporting System, 2010-2017

**DOI:** 10.1001/jamanetworkopen.2019.9249

**Published:** 2019-08-14

**Authors:** Graça M. Dores, Marthe Bryant-Genevier, Silvia Perez-Vilar

**Affiliations:** 1 US Food and Drug Administration, Center for Biologics Evaluation and Research, Office of Biostatistics and Epidemiology, Division of Epidemiology, Silver Spring, Maryland

## Abstract

**Importance:**

Sipuleucel-T was the first therapeutic cancer vaccine approved by the US Food and Drug Administration (FDA) in 2010. Although almost a decade has passed since its approval for the treatment of asymptomatic or minimally symptomatic castration-resistant prostate cancer (CRPC), there remains a paucity of literature describing safety data in the postmarketing period.

**Objective:**

To describe the postmarketing safety experience for sipuleucel-T.

**Design, Setting, and Participants:**

In this case series study, US reports for sipuleucel-T submitted to the FDA’s Adverse Event Reporting System were searched and reviewed between April 29, 2010, and December 31, 2017. This system is a spontaneous safety surveillance database for drug and therapeutic biologic products. The analysis of 3216 reports and select case reviews were undertaken between February and November 2018.

**Main Outcomes and Measures:**

Descriptive statistics were used to assess adverse event reports for sipuleucel-T. Empirical Bayes Geometric Means (EBGM) and their 90% confidence intervals (CIs) were computed to identify disproportionate (ie, at least twice the expected) reporting of sipuleucel-T–event pairs. Selected adverse events and death reports were individually reviewed.

**Results:**

In total, 3216 reports were identified for sipuleucel-T, of which 2014 (62.6%) were serious. For all included reports, the patients’ median (interquartile range) age was 73 (67-79) years, and 3149 were specified to be males. Chills (n = 318), malaise (n = 196), pyrexia (n = 189), culture positive (n = 184), fatigue (n = 180), and nausea (n = 173) were among the most commonly reported adverse events. Infusion-related reactions (EBGM, 12.1; 90% CI, 9.4-15.3), infections, vascular events, and transient ischemic attacks (EBGM, 2.9; 90% CI, 2.2-3.9) were reported disproportionately. Among 249 deaths for which relevant dates were available, 128 (51.4%) were reported within 30 days of a sipuleucel-T infusion, of which 81.2% included a specified cause of death; of these 104 deaths, there were 37 neoplasms (35.6%), 25 cardiac disorders (24.0%), 18 nervous system disorders (17.3%), and 9 infections (8.7%).

**Conclusions and Relevance:**

Reported adverse events were generally consistent with the safety experience observed in prelicensure studies and described in the sipuleucel-T package insert. Off-label use among overtly symptomatic men with CRPC, reporting bias, or lack of product effectiveness may have influenced the reporting of deaths within 30 days of treatment initiation. With this overview of sipuleucel-T experience, the present study serves as a resource for health care professionals and patients as they weigh the risks and benefits of treatment in the context of all available therapeutic options for CRPC.

## Key Points

**Question:**

What is the postmarketing safety profile of sipuleucel-T in the United States?

**Findings:**

The US Food and Drug Administration’s Adverse Event Reporting System received 3216 reports for sipuleucel-T from 2010 through 2017. This case series study identified disproportionate reporting for infusion-associated reactions, infections, certain thromboembolic events, and transient ischemic attacks.

**Meaning:**

The spectrum of adverse events reported for sipuleucel-T was consistent with the safety experience described in clinical studies and the package insert, and no new safety concerns were identified.

## Teaser

This case series uses data from the US Food and Drug Administration’s Adverse Event Reporting System to assess the postmarketing safety profile of sipuleucel-T.

## Introduction

Sipuleucel-T (Provenge), an autologous active cellular immunotherapy, was the first therapeutic cancer vaccine approved by the US Food and Drug Administration (FDA) (April 29, 2010) for the treatment of asymptomatic or minimally symptomatic metastatic, castration-resistant prostate cancer (CRPC). The safety database leading to product approval included 904 patients (601 patients treated with sipuleucel-T; 303 controls) from 4 randomized, placebo-controlled clinical trials.^1,2^ The most common adverse events (AEs) among patients treated with sipuleucel-T were chills, fatigue, fever, back pain, nausea, arthralgia, and headache. Serious AEs occurred in 24% of treated men and 25% of controls.^1^ However, an imbalance in the occurrence of cerebrovascular events (3.5% of patients treated with sipuleucel-T vs 2.6% of controls) led the US FDA to require the sponsor to conduct the observational phase IV study Provenge Registry for Observation, Collection, and Evaluation of Experience Data (PROCEED) to further quantify the risk of cerebrovascular events, including hemorrhagic/ischemic strokes and transient ischemic attacks (TIAs), following sipuleucel-T therapy.^3^ The trial^4^ was closed to patient accrual, and the results published in the peer-reviewed literature are awaited.

As of 2019, 30 000 men have been prescribed sipuleucel-T.^5^ With few exceptions, including 1 institutional retrospective review (36 patients treated with sipuleucel-T) and a clinical trial of androgen-dependent prostate cancer (117 patients treated with sipuleucel-T),^6,7^ most of the safety information in the literature^8,9,10,11^ is limited to review articles that refer to the clinical trials originally submitted in support of product approval.^12,13,14^ We sought to summarize the safety experience of sipuleucel-T in the postmarketing period by assessing US reports submitted to the FDA’s Adverse Event Reporting System (FAERS).

## Methods

### FAERS Database

We used the FAERS database to assess AEs reported for sipuleucel-T; FAERS is a spontaneous, passive surveillance system designed for health care professionals, pharmaceutical companies, and consumers to report AEs, medication errors, and product quality matters associated with drugs and biologic products.^15^ The database includes information on patient demographics, medical history, concomitant medications, description and outcome of the AE, and source of the report. All AEs are coded according to the international Medical Dictionary for Regulatory Activities.^16,17^ These coded terms are arranged in a hierarchy of 5 categories that include broad (system organ class [SOC]) and specific categories (eg, preferred term [PT]). A PT can describe signs or symptoms, a diagnosis, an indication for treatment, laboratory tests, procedures, and aspects of medical, social, or family history.^16^ A single FAERS report is assigned 1 or more PTs and the corresponding SOC(s) for each PT. Each PT is considered independently of any other. A report is considered serious when the patient outcome results in death, life-threatening illness, hospitalization or prolongation of existing hospitalization, permanent disability, or birth defect.^18^ The FAERS reports are submitted as expedited (15-day) reports (reports for both serious and unexpected [unlabeled] AEs required to be submitted by industry to the US FDA within 15 days of receipt), direct reports (any report submitted directly to the US FDA from outside of industry, eg, health care professionals or consumers), or nonexpedited (periodic) reports (reports for serious labeled events and nonserious events) submitted by industry to the US FDA on a regular basis. This safety review was exempt from institutional review board approval or informed patient consent because it met the criteria for exemption from the Office for Protection From Research Risks, as specified under Department of Health and Human Services regulations.^19^ This report followed the reporting guideline for case series.

### Eligibility Criteria

We searched US reports submitted to FAERS between April 29, 2010, and December 31, 2017, with sipuleucel-T included as a suspect product. A suspect product is a drug or biologic considered by the reporter to be associated with the reported AE. We excluded 2 reports involving females and 8 duplicate or invalid reports encountered during the manual case review.

### Statistical Analysis

#### Descriptive Analyses

We separately assessed all reports (serious and nonserious) and serious reports only. Overall frequencies and stratified analyses by seriousness were undertaken using Stata/IC 13.1 (StataCorp LLC). Case review and analyses were conducted between February and November 2018.

#### Data Mining (Disproportionality Analyses)

We used the Multi-Item Gamma Poisson Shrinker algorithm in the Oracle Empirica Signal system to conduct empirical Bayes data mining to assess disproportionality in AE reporting for sipuleucel-T compared with all other therapeutic biologics and drugs in the FAERS database.^20,21,22^ The analyses (data lock point, February 2, 2018) were adjusted for age group (≤1, 2-4, 5-12, 13-16, 17-29, 30-45, 46-64, 65-75, 76-85, and ≥86 years and unknown age) and calendar year in which the report was received at the US FDA. The main statistical scores computed were the empirical Bayes Geometric Mean (EBGM) and the 90% confidence interval (EB05, EB95). An elevated data mining statistic should be interpreted as a potential signal for further evaluation.^21,23^ The EBGM does not reflect the magnitude of association between sipuleucel-T and an AE but provides an estimate of the relative reporting of an event for sipuleucel-T relative to all other drugs and events in the FAERS database. An EB05 of 2.0 or higher is commonly used by the US FDA as a criterion for considering an AE a potential signal because such a value suggests a high probability of the product-event pair occurring at least twice as often as expected under the assumption that product-events are randomly paired.^21^ The analysis performed in Empirica relies on the most recent follow-up report for a given case and on a duplicate detection algorithm (the most recent follow-up serves to represent the group). Therefore, the number of reports in the FAERS database may differ slightly from those available for data mining analyses.

### Case Reviews

On the basis of findings from data mining analyses and premarketing experience, we reviewed reports for selected AEs, including, but not limited to, thromboembolic and vascular events such as deep vein thrombosis (DVT), pulmonary embolism (PE), TIA, stroke, and myocardial infarction (MI).^24^ We also reviewed all death reports (n = 314) to identify the stated cause of death; when this information was not available, we assigned a cause of death if apparent in the case history. We calculated time to death from the date of last sipuleucel-T infusion. For cases with missing day of infusion or death, we imputed the day as 15. If only the calendar year was available, we imputed the month as June; if partial information was available, we used the month midway between the known month of infusion (or death), using the earliest possible month for the unknown month of infusion or latest possible month for the unknown month of death. Overall, we imputed dates on 39 death reports (12.4% of all death reports).

## Results

We identified a total of 3216 reports following sipuleucel-T administration, of which 2014 (62.6%) were serious (Table 1). The median (interquartile range [IQR]) patient age was 73 (67-79) years, and 3149 patients were specified to be males. Most reports were submitted by physicians or other health care professionals; some reports involved patients enrolled in clinical trials or in the postmarketing PROCEED study.^1,4^

**Table 1.** Description of US Reports Submitted to the US FDA Adverse Event Reporting System for Sipuleucel-T Between April 29, 2010, and December 31, 2017

Report	No. (%)	
	All Reports	Serious Reports Only
Total	3216 (100)	2014 (100)
Sex		
Male	3149 (97.9)	1952 (96.9)
Not specified	67 (2.1)	62 (3.1)
Age, y		
<50	12 (0.4)	4 (0.2)
50-64	448 (13.9)	286 (14.2)
65-74	1065 (33.1)	696 (34.5)
75-84	860 (26.7)	582 (28.9)
≥85	249 (7.7)	174 (8.6)
Not specified	582 (18.1)	272 (13.5)
Received at FDA, calendar year		
2010	44 (1.4)	44 (2.2)
2011	203 (6.3)	199 (9.9)
2012	317 (9.9)	309 (15.3)
2013	599 (18.6)	390 (19.4)
2014	261 (8.1)	254 (12.6)
2015	294 (9.1)	279 (13.9)
2016	665 (20.7)	256 (12.7)
2017	833 (25.9)	283 (14.1)
Source of report		
Health professional	1426 (44.3)	920 (45.7)
Physician	1077 (33.5)	712 (35.4)
Consumer/other specified	547 (17.0)	276 (13.7)
Registered nurse	101 (3.1)	72 (3.6)
Pharmacist	43 (1.3)	24 (1.2)
Not specified	22 (0.7)	10 (0.5)
Type of report		
Expedited, 15 d	1586 (49.3)	1573 (78.1)
Not expedited	1601 (49.8)	412 (20.5)
Direct	29 (0.9)	29 (1.4)
Reported outcome, serious reports		
Death	314 (9.8)	314 (15.6)
Nonfatal: hospitalization, disability, life-threatening, or required intervention	1099 (34.2)	1099 (54.6)
Nonfatal: other serious outcomes^a^	600 (18.7)	600 (29.8)
Not specified	1 (<0.1)	1 (<0.1)
Not applicable^b^	1202 (37.4)	0

Abbreviation: FDA, Food and Drug Administration.

^a^ Defined as important medical events based on appropriate medical judgment that may jeopardize the patient or participant and may require medical or surgical intervention to prevent one of the other specified outcomes.

^b^ Refers to nonserious reports. Reports that do not meet criteria for seriousness as defined in the Code of Federal Regulations^18^ (ie, death, hospitalization, life-threatening, disability, congenital anomaly, required intervention, or other serious outcome) are considered nonserious.

Of 3216 reports, 9800 PTs were included. The most frequently reported PTs included chills (n = 318), malaise (n = 196), pyrexia (n = 189), culture positive (n = 184), fatigue (n = 180), and nausea (n = 173) (Table 2). Except for culture positive, these PTs were also frequent among serious reports in addition to disease progression (potentially confounded by the indication for therapy) and asthenia.

**Table 2.** Top 10 Ranked PTs for US Adverse Event Reports Submitted to the US FDA Adverse Event Reporting System for Sipuleucel-T, Overall and According to Seriousness, April 29, 2010, to December 31, 2017

PT	No. (Rank)	
	All Reports	Serious Reports Only
Total	9800	7488
Chills	318 (1)	166 (1)
Malaise	196 (2)	117 (5)
Pyrexia	189 (3)	118 (4)
Culture positive	184 (4)	Ranked >10
Fatigue	180 (5)	118 (4)
Nausea	173 (6)	110 (8)
Disease progression^a^	147 (7)	138 (2)
Asthenia	144 (8)	120 (3)
Ill-defined disorder	138 (9)	Ranked >10
Dizziness	124 (10)	Ranked >10
Anemia	Ranked >10	115 (6)
Hospitalization	Ranked >10	111 (7)
Fall	Ranked >10	101 (9)
Dehydration	Ranked >10	95 (10)

Abbreviations: FDA, Food and Drug Administration; PTs, preferred terms.

^a^ Confounded by the indication for sipuleucel-T therapy.

### Data Mining (Disproportionality Analyses)

Sipuleucel-T–event pairs reported at least twice as often as expected (EB05 ≥ 2.0) are provided in Table 3. We observed disproportionate reporting for labeled PTs, including vomiting (EBGM, 2.7; 90% CI, 2.4-3.1), chills (EBGM, 15.8; 90% CI, 14.4-17.3), pyrexia (EBGM, 4.5; 90% CI, 4.0-5.1), culture positive (EBGM, 157.1; 90% CI, 138.8-177.4), infusion-related reaction (EBGM, 12.1; 90% CI, 9.4-15.3), and citrate toxicity (EBGM, 153.1; 90% CI, 99.1-228.1). We also identified anemia (EBGM, 3.0; 90% CI, 2.6-3.5), infections (eg, sepsis [EBGM, 2.8; 90% CI, 2.3-3.4], bacteremia [EBGM, 7.6; 90% CI, 5.2-11.6]), device-associated infections (eg, device-related infection [EBGM, 29.9; 90% CI, 25.5-34.8] and device-related sepsis [EBGM, 48.9; 90% CI, 33.2-69.9]), and urinary tract infection [EBGM, 3.8; 90% CI, 3.2-4.5]), presyncope (EBGM, 3.6; 90% CI, 2.5-5.1), blood pressure decrease (EBGM, 3.6; 90% CI, 2.8-4.5), tachycardia (EBGM, 4.8; 90% CI, 3.7-6.1), unresponsive to stimuli (EBGM, 3.3; 90% CI, 2.4-4.5), and thromboembolic events (eg, PE [EBGM, 3.1; 90% CI, 2.4-3.8], thrombosis in device [EBGM, 27.7; 90% CI, 19.3-38.7], DVT [EBGM, 3.2; 90% CI, 2.6-4.0], and jugular vein thrombosis [EBGM, 16.9; 90% CI, 4.1-45.0]) as reported disproportionately.

**Table 3.** Data Associated With PTs With a Disproportionality EB05 Score of 2.0 or Higher Grouped by SOC for US Adverse Event Reports Submitted to the US FDA Adverse Event Reporting System for Sipuleucel-T, Overall and According to Seriousness, April 29, 2010, to December 31, 2017

SOC and PT	All Reports^a^^,^^b^		Serious Reports Only		Labeled Event^c^
	No.	EBGM (90% CI)	No.	EBGM (90% CI)	
Blood and lymphatic system disorders					
Anemia	125	3.0 (2.6-3.5)	119	2.9 (2.5-3.3)	Yes
Leukocytosis	15	4.1 (2.7-6.0)	14	3.4 (2.2-5.1)	No
Normochromic normocytic anemia	6	6.5 (2.7-26.6)	6	5.1 (2.4-17.0)	Yes
Anemia of chronic disease	6	4.9 (2.4-12.2)	6	4.4 (2.3-9.1)	Yes
Cardiac disorders^d^					
Tachycardia	42	4.8 (3.7-6.1)	33	3.7 (2.8-4.9)	Yes
Sinus tachycardia	8	3.8 (2.1-6.3)	EB05 < 2.0	EB05 < 2.0	Yes
Gastrointestinal disorders					
Vomiting	140	2.7 (2.4-3.1)	97	2.7 (2.3-3.2)	Yes
Oral paresthesia	9	4.2 (2.5-6.9)	EB05 < 2.0	EB05 < 2.0	Yes
Nausea	Lower score^e^	Lower score^e^	125	2.4 (2.1-2.8)	Yes
General disorders and administration site conditions					
Chills	317	15.8 (14.4-17.3)	166	13.0 (11.4-14.8)	Yes
Malaise	192	3.1 (2.8-3.5)	114	3.3 (2.8-3.8)	No
Pyrexia	191	4.5 (4.0-5.1)	120	3.5 (3.0-4.0)	Yes
Feeling cold	16	3.1 (2.1-4.6)	EB05 < 2.0	EB05 < 2.0	Yes
Infusion site extravasation	14	8.2 (4.6-17.1)	EB05 < 2.0	EB05 < 2.0	Yes
SIRS	11	8.0 (4.1-19.3)	11	6.8 (3.7-16.0)	No
Complication associated with device	10	4.0 (2.4-6.4)	EB05 < 2.0	EB05 < 2.0	Yes
Infusion site pain	7	3.9 (2.1-6.6)	EB05 < 2.0	EB05 < 2.0	Yes
Infusion site swelling	6	4.4 (2.3-8.4)	EB05 < 2.0	EB05 < 2.0	Yes
Catheter site pain	5	4.5 (2.1-12.0)	EB05 < 2.0	EB05 < 2.0	Yes
Infusion site hematoma	5	28.4 (3.8-82.8)	EB05 < 2.0	EB05 < 2.0	Yes
Infusion site reaction	5	5.9 (2.3-28.7)	EB05 < 2.0	EB05 < 2.0	Yes
Infections and infestations					
Device-related infection	110	29.9 (25.5-34.8)	94	24.2 (20.4-28.6)	Yes
Urinary tract infection	88	3.8 (3.2-4.5)	70	3.3 (2.7-4.0)	Yes
Infection	64	3.4 (2.7-4.1)	EB05 < 2.0	EB05 < 2.0	Yes
Sepsis	62	2.8 (2.3-3.4)	62	2.6 (2.1-3.2)	Yes
Bacteremia	24	7.6 (5.2-11.6)	23	6.6 (4.6-9.6)	Yes
Staphylococcal bacteremia	22	22.5 (15.5-31.8)	22	19.6 (13.4-27.8)	Yes
Device-related sepsis	20	48.9 (33.2-69.9)	20	42.9 (29.2-61.3)	Yes
Catheter site infection	10	5.2 (3.1-8.6)	EB05 < 2.0	EB05 < 2.0	Yes
Staphylococcal sepsis	8	4.7 (2.6-8.0)	8	4.2 (2.4-7.0)	Yes
Injury, poisoning, and procedural complications					
Infusion-related reaction	65	12.1 (9.4-15.3)	47	9.3 (6.9-12.6)	Yes
Citrate toxicity	16	153.1 (99.1-228.1)	11	130.0 (76.4-209.9)	Yes
Investigations					
Culture positive	180	157.1 (138.8-177.4)	50	111.5 (87.8-140.0)	Yes
Hemoglobin decreased	91	3.4 (2.9-4.1)	74	2.8 (2.3-3.4)	Yes
Blood pressure decreased	48	3.6 (2.8-4.5)	34	3.4 (2.5-4.4)	Yes
Mononuclear cell count abnormal	39	161.6 (123.1-209.1)	5	71.4 (29.9-149.7)	No^e^
Hematocrit decreased	33	5.4 (4.1-7.1)	30	4.8 (3.5-6.4)	Yes
Body temperature increased	32	10.0 (6.8-14.9)	24	8.2 (5.4-13.3)	Yes
Microbiology test abnormal	21	154.3 (105.9-218.6)	EB05 < 2.0	EB05 < 2.0	Yes
*Staphylococcus* test positive	13	12.3 (5.4-24.5)	12	7.5 (4.0-17.2)	Yes
Neutrophil count abnormal	7	5.7 (2.8-17.2)	EB05 < 2.0	EB05 < 2.0	No
Blood culture positive	6	4.3 (2.2-7.8)	6	3.9 (2.0-6.9)	Yes
Gram stain positive	4	17.0 (2.4-87.7)	EB05 < 2.0	EB05 < 2.0	Yes
Metabolism and nutrition disorders					
Dehydration	106	3.1 (2.6-3.6)	93	2.7 (2.3-3.2)	No
Hyponatremia	28	3.9 (2.8-5.2)	28	3.5 (2.6-4.8)	No
Hypokalemia	22	4.3 (3.0-6.0)	22	3.9 (2.7-5.4)	No
Malnutrition	12	4.1 (2.5-6.3)	12	3.8 (2.4-5.7)	No
Hypovolemia	11	4.2 (2.6-6.6)	11	3.8 (2.4-5.9)	No
Musculoskeletal and connective tissue disorders					
Back pain	111	2.8 (2.4-3.2)	69	2.9 (2.4-3.6)	Yes
Bone pain	32	3.9 (2.9-5.2)	24	4.6 (3.3-6.3)	Yes
Nervous system disorders^f^					
Tremor	66	2.6 (2.1-3.2)	EB05 < 2.0	EB05 < 2.0	Yes
Transient ischemic attack	30	2.9 (2.2-3.9)	EB05 < 2.0	EB05 < 2.0	Yes
Unresponsive to stimuli	25	3.3 (2.4-4.5)	24	2.9 (2.0-3.9)	No
Presyncope	20	3.6 (2.5-5.1)	17	3.4 (2.3-4.9)	Yes
Carotid artery stenosis	7	3.8 (2.1-6. 6)	EB05 < 2.0	EB05 < 2.0	No
Product					
Thrombosis in device	23	27.7 (19.3-38.7)	11	6.3 (3.5-14.0)	Yes
Device occlusion	18	7.3 (4.7-12.4)	EB05 < 2.0	EB05 < 2.0	Yes
Product contamination microbial	7	8.6 (3.2-30.1)	EB05 < 2.0	EB05 < 2.0	Yes
Product sterility lacking	5	44.7 (5.9-106.6)	5	36.3 (4.6-91.5)	Yes
Renal and urinary disorders					
Hematuria	48	3.1 (2.4-3.9)	46	3.2 (2.5-4.0)	Yes
Respiratory, thoracic, and mediastinal disorders					
Pulmonary embolism	52	3.1 (2.4-3.8)	52	2.7 (2.2-3.4)	Yes
Atelectasis	23	6.2 (4.4-8.7)	23	5.3 (3.8-7.4)	No
Hypoxia	22	2.9 (2.1-4.1)	EB05 < 2.0	EB05 < 2.0	Yes
Surgical and medical procedures					
Hospitalization	105	5.1 (4.3-5.9)	105	4.6 (3.9-5.4)	Outcome
Hospice care	15	5.0 (3.3-7.5)	10	3.8 (2.3-6.1)	Outcome
Vascular disorders					
Deep vein thrombosis	53	3.2 (2.6-4.0)	53	2.9 (2.3-3.6)	Yes
Pallor	30	7.5 (5.4-10.4)	18	4.9 (3.3-7.0)	No
Hematoma	26	3.6 (2.6-4.9)	EB05 < 2.0	EB05 < 2.0	Yes
Poor venous access	22	36.0 (24.9-50.6)	8	26.6 (7.9-50.9)	Yes
Thrombophlebitis superficial	8	6.1 (3.1-16.9)	8	5.5 (2.9-13.9)	Yes
Jugular vein thrombosis	7	16.9 (4.1-45.0)	7	13.6 (3.6-39.0)	Yes

Abbreviations: EBGM, empirical Bayes Geometric Mean; EB05, lower bound of EBGM 90% CI; FDA, Food and Drug Administration; PT, preferred term; SIRS, systemic inflammatory response syndrome; SOC, system organ class.

^a^ The following PTs (all reports) within the specified SOC had an EB05 of 2.0 or higher but were not included in the table because there is potential confounding by the following indications. General disorders and administration site conditions: disease progression (n = 139); investigations: prostate-specific antigen increased (n = 66), blood alkaline phosphate increased (n = 23); neoplasms: prostate cancer (n = 52), prostate cancer metastatic (n = 49), metastases to bone (n = 45), metastases to central nervous system (n = 20), neoplasm progression (n = 18), metastases to liver (n = 17), metastasis (n = 13), metastases to spine (n = 7), metastases to lymph nodes (n = 6), and metastases to meninges (n = 6); nervous system disorders: spinal cord compression (n = 23); and renal disorders: hydronephrosis (n = 24), urinary tract obstruction (n = 8), ureteric obstruction (n = 7), bladder obstruction (n = 7). Although back pain, bone pain, and hematuria could also be associated with confounding by indication, these symptoms and signs are also common in conditions unrelated to prostate cancer; therefore, we maintained these entities in the table.

^b^ The following PTs (all reports) within the specified SOC had an EB05 of 2.0 or higher but were not included in the table owing to nonspecific terminology. General disorders and administration site conditions: ill-defined disorder (n = 137), adverse reaction (n = 11); investigations: investigation (n = 8).

^c^ Indicates whether the adverse event (represented by the specified PT) is a labeled event or consistent with a labeled event. The product label instructs patients to report catheter-associated symptoms (eg, swelling, redness) to a health care professional; therefore, we considered all device- or catheter-associated events as labeled events, including hematoma.

^d^ Although the EB05 was lower than 2.0, reports of myocardial infarction (n = 38) were reviewed owing to the inclusion of cardiovascular disorders in the “Warnings and Precautions” section of the product label (EBGM [serious reports], 0.53; 90% CI, 0.40-0.68).

^e^ Not a labeled event with respect to peripheral blood; labeled event with respect to characterization of product.

^f^ Although EB05 was lower than 2.0, reports of stroke were reviewed owing to the inclusion of cerebrovascular disease in the “Warnings and Precautions” section of the product label. Data for all serious reports were as follows: ischemic stroke (n = 6; EBGM, 1.7; 90% CI, 0.9-3.0); hemorrhagic stroke (n = 5; EBGM, 1.3; 90% CI, 0.7-2.3); embolic stroke (n = 6; EBGM, 2.9; 90% CI, 1.5-5.0). All reports were serious.

We observed disproportionate reporting for local infusion site signs and symptoms, including extravasation, hematoma, swelling, and reaction when all (serious and nonserious) reports were considered. This pattern was similarly observed for TIA, tremor, oral paresthesia (the latter likely reflecting citrate toxicity associated with leukapheresis), and product- or device-associated AEs, such as microbial product contamination, device occlusion, catheter site pain, catheter site infection, and complication associated with device. In addition, the data mining analyses detected disproportionate reporting for AEs not described in the US package insert (USPI), including leukocytosis, malaise, systemic inflammatory response syndrome (SIRS), abnormal mononuclear cell count, dehydration, hyponatremia, hypokalemia, malnutrition, hypovolemia, atelectasis, and pallor.

### Case Review

#### Death Reports

Of 314 reported deaths, the median (IQR) age of patients was 74 (68-81) years. Most deaths occurred in the age groups of 65 to 74 years (106 patients [33.8%]) and 75 to 84 years (95 patients [30.3%]) (Table 4). There were 249 reports (79%) for which the time interval from last sipuleucel-T infusion and death was available or could be imputed (39 [12.4%]). More than half the deaths (128 [51.4%]) occurred within 30 days of a sipuleucel-T infusion, an additional 59 deaths (23.7%) between 31 and 90 days, and 42 deaths (16.9%) after 180 days (Table 4). Similar proportions (53.8%, 23.3%, and 16.2%, respectively) were estimated when only reports with known dates were considered (n = 210). Most death reports were attributable to neoplasms (83 [33.3%]), cardiac disorders (38 [15.3%]), nervous system disorders (33 [13.3%]), and infections and infestations (14 [5.6%]). Among the 128 deaths reported within 30 days of a sipuleucel-T infusion, 104 patients (81.2%) had a specified cause of death; of these 104 deaths, 37 (35.6%) were associated with neoplasms, followed by cardiac disorders (25 [24.0%]), nervous system disorders (18 [17.3%]), and infection (9 [8.7%]).

**Table 4.** Characterization of Deaths for US Adverse Event Reports Submitted to the US FDA Adverse Event Reporting System for Sipuleucel-T, April 29, 2010, to December 31, 2017

Characteristic	Total, No (%)
Total death reports	314 (100)
Total death reports with date of last sipuleucel-T infusion and date of death	
Without imputation	210 (66.9)
With imputation	249 (79.3)
Age, y^a^	
<50	1 (0.3)
50-64	31 (9.9)
65-74	106 (33.8)
75-84	95 (30.3)
≥85	32 (10.2)
Not specified	49 (15.6)
Time from last infusion to death, d^b^	
<31	128 (51.4)
31-90	59 (23.7)
91-180	20 (8.0)
>180	42 (16.9)
Cause of death according to SOC^b^	
Total deaths^c^	
Neoplasms	83 (33.3)
Cardiac disorders	38 (15.3)
Nervous system disorders	33 (13.3)
Infections and infestations	14 (5.6)
General	8 (3.2)
Respiratory tract	7 (2.8)
Vascular	4 (1.6)
Renal	3 (1.2)
Other specified	5 (2.0)
Not specified	54 (21.7)
Deaths occurring within 30 d of sipuleucel-T infusion^c^	
Neoplasm	37 (28.9)
Cardiac	25 (19.5)
Neurologic	18 (14.1)
Infections and infestations	9 (7.0)
General	3 (2.3)
Respiratory tract	5 (3.9)
Vascular	3 (2.3)
Other specified	4 (3.1)
Not specified	24 (18.8)

Abbreviations: FDA, US Food and Drug Administration; SOC, system organ class.

^a^ Based on total deaths (n = 314).

^b^ Based on 249 deaths (with available dates of death and last sipuleucel-T infusion, including imputed dates).

^c^ Causes of death associated with fewer than 3 cases were grouped in the “other specified” category to maintain patient anonymity.

#### Tachycardia

Among 33 serious reports of tachycardia (median [IQR] age, 79 [72-85] years; n = 32), the median time to onset from last sipuleucel-T infusion was 8 days (IQR, 0-13 days, with day 0 representing events occurring on the day of infusion; n = 29). Twelve reports (36.0%) were associated with an infusion reaction occurring within 1 day of infusion. Four reports of tachycardia were potentially associated with underlying heart disease (new-onset atrial fibrillation, chest pain, acute MI, and ischemia). The remaining reports were associated with fever and infection (n = 9) or dehydration (n = 5).

#### Myocardial Infarction

There were 38 reports (all serious) of MI, of which 2 were excluded from these descriptive analyses based on MI occurring prior to sipuleucel-T infusion or the diagnosis being ruled out, as described in the report. The median (IQR) age of the remaining 35 patients was 74 (71-81) years, and 1 patient was an unspecified age. Cardiac risk factors (eg, hypertension, diabetes, coronary artery disease, or hyperlipidemia) were specified in 68% of reports. Most events occurred after the second (n = 14) or third (n = 11) dose of sipuleucel-T; 6 events occurred after the first dose, and 5 reports did not specify the timing of subsequent MI. Most MIs occurred within 1 week of a sipuleucel-T infusion (19 [53%], with 12 of these reports describing MI on the same day as an infusion) or 8 to 30 days after an infusion (n = 11).

#### Transient Ischemic Attack

We identified a total of 30 (serious and nonserious) reports of TIA following at least 1 dose of sipuleucel-T. The median (IQR) age at time of the event was 75 (67-79) years, and the median (range) time to the event, which was assessable in all but 4 reports, was 5 (0-11) days.

#### Stroke

All 17 reports of stroke (6 embolic, 5 hemorrhagic, and 6 ischemic) occurring among 16 patients were serious. One patient was diagnosed as having an embolic stroke 6 days after his second dose of sipuleucel-T and an ischemic stroke 15 days later. Stroke occurred after the first (n = 3), second (n = 4), or third (n = 7; includes 5 patients in the PROCEED study) sipuleucel-T infusion. Overall, the median (IQR) time from the last sipuleucel-T dose to stroke was 25 (6-206) days among 14 patients with information. The median (IQR) time to stroke was 7 (0-14) days among those not stated to be in the study and 490 days (range, 206-996 days) among those participating in the PROCEED study.

#### Other Thromboembolic Events

Forty-one of the 53 DVT reports (77%) and 47 of the 52 PE reports (90%) occurred within 30 days of a sipuleucel-T infusion. All 7 cases of jugular vein thrombosis occurred in association with a central venous catheter (CVC).

#### Systemic Inflammatory Response Syndrome

There were 11 serious reports of SIRS submitted between 2012 and 2015 that occurred after the first, second, or third sipuleucel-T infusion. The median (IQR) age of these patients was 78 (67-86) years, and the median (IQR) time from infusion to SIRS was 0 (0-11) days. Nine reports documented administration of intravenous antibiotics and included other PTs, such as sepsis, bacteremia, bacterial infection, pneumonia, catheter site infection, and device-related sepsis, suggesting an infectious cause leading to SIRS. This syndrome was also reported in conjunction with infusion reaction, congestive heart failure, and PE.

#### Unresponsive to Stimuli

Serious reports of unresponsive to stimuli (n = 23) were associated with preterminal events (n = 7; eg, progression of CRPC, intracerebral bleed, or cardiopulmonary arrest), infusion reactions (n = 6), falls (n = 3), and other isolated cases of seizure, TIA, drug reaction (other than to sipuleucel-T), infection, and illicit drug use. Five of the infusion reactions occurred on the day of sipuleucel-T receipt during or shortly following the infusion (1 case did not specify event timing). Two reports described unresponsive events during the leukapheresis procedure.

#### Other AEs

Leukocytosis was reported in association with infection, infusion reaction, progression of metastatic disease, thrombosis, subdural hematoma, and acute kidney injury or obstruction. Malaise, hyponatremia, hypokalemia, malnutrition, hypovolemia, and atelectasis were secondary manifestations of various underlying labeled disease processes and also associated with progression of metastatic prostate cancer, the indication for therapy. There were 5 serious reports involving patients unable to undergo sipuleucel-T infusion on the scheduled date because their mononuclear cell counts were outside the required range (sipuleucel product).

## Discussion

The present study represents the largest postmarketing safety assessment, to our knowledge, of sipuleucel-T since its entry in the US market in 2010. The reported AEs were generally consistent with the safety experience observed in prelicensure studies and described in the USPI.^12,13,14,24^ Except for asthenia, dizziness, and fall, the most frequently reported AEs (chills, malaise, pyrexia, and culture positive) were also identified in our data mining analyses. Fall, typically an accidental event, is not included in the USPI, whereas asthenia, dizziness, and fatigue are labeled events.^24^ We found that more than 50% of reported deaths occurred within 30 days of a sipuleucel-T infusion, although, based on the information available in the FAERS reports, an association between death and sipuleucel-T could not be established. In data mining analyses, we identified disproportionate reporting (at least twice the expected) for anemia, infection, thromboembolic events, and general symptoms, all of which (or related term) are included in the USPI.

Various metabolic abnormalities and SIRS represent unlabeled events reported more frequently than expected. However, metabolic abnormalities and SIRS were noted to be secondary manifestations of labeled events, most commonly infections and infusion reactions. A nonspecific clinical response, SIRS reflects the manifestation of a dysregulated inflammatory response triggered by infectious or noninfectious conditions.^25,26^ The infusion of sipuleucel-T is associated with an immunostimulatory response that could conceivably result in a dysregulated inflammatory response. The majority of SIRS events occurred within 1 day of infusion, with most SIRS reports reflecting an underlying infection or infusion reaction. In the literature, the term *SIRS* has been found to lack specificity and sensitivity and has largely been replaced by alternate terminology.^27,28,29^ This may explain why FAERS reports with a SIRS diagnosis were not observed after 2015. Other unlabeled AEs that were primarily noted in serious reports included pallor, unresponsive to stimuli, dehydration, malaise, and leukocytosis. Pallor would be an expected clinical sign of anemia, the latter a recognized and labeled sequela of sipuleucel-T that also was disproportionately reported. Unresponsive to stimuli mostly described preterminal events and infusion reactions. Similar to dehydration and malaise, leukocytosis reflected other underlying conditions.

Infections were among the most notable disproportionately reported AEs, with infections associated with CVCs among the most frequently reported. Catheter-associated infections have been previously reported to occur in 12% of patients treated with sipuleucel-T (64% severe or life-threatening) in contrast to the 1% observed among those without a CVC.^30^ Although the frequency of CVC infection appears elevated compared with that generally seen in other settings,^31,32^ comparisons across studies are limited because of varying study methods, measures of association, indications for CVCs, duration of use, and patient populations.

Thromboembolic events, including TIA, PE, thrombosis in device, and jugular vein thrombosis, were also disproportionately reported. Prostate cancer, advancing age, androgen-deprivation therapy and its duration, advanced cancer stage, and tumor burden are risk factors for thrombosis (including cerebrovascular disease)^33,34,35^; these risks could have contributed to the thromboembolic events observed after administration of sipuleucel-T. Although TIA was reported disproportionately, PTs for cerebrovascular accident or stroke were not. We found a long latency period for stroke among reports indicating patient participation in the PROCEED study (median, 490 days) in contrast to those not specifying trial participation (median, 7 days). In the preapproval study by Kantoff and colleagues,^13^ a median follow-up of 34.1 months was reported, and the median time to cerebrovascular events was 210 days. However, reporting sensitivity for cerebrovascular events may have been heightened among clinical trial participants with active follow-up and in the postmarketing PROCEED study, during which events were actively solicited. It is possible that cerebrovascular accidents and strokes may have been underreported to FAERS if they had a long latency period and were not recognized as possibly being associated with sipuleucel-T.

A few deaths (0.5%) were observed within 30 days of receiving sipuleucel-T in the preapproval trials, during which most deaths occurred more than 1 year after treatment.^2^ In the present study, we found that prostate cancer accounted for most deaths occurring within 30 days of a sipuleucel-T infusion, possibly reflecting increased sensitivity of passive surveillance systems for events occurring in close proximity to the exposure,^36,37,38^ patients not meeting labeled indications for treatment, or lack of product effectiveness. Several treatment alternatives for metastatic CRPC became available after sipuleucel-T approval in 2010.^39^ Therefore, after 2010, patients may have been more heavily pretreated, thereby presenting for sipuleucel-T treatment with more refractory or symptomatic disease and an ensuing shorter survival time compared with individuals participating in the preapproval clinical trials. However, a recent study using a US insurance database found that 69% of patients received sipuleucel-T as first-line treatment, suggesting that many patients receiving sipuleucel-T are naive to other therapies for CRPC.^39^ In the present study, cardiac death was the second most common cause of death, although cardiac-related AEs (eg, MI, arrhythmia) were not disproportionately reported except for tachycardia, which was generally a secondary phenomenon.

### Strengths and Limitations

An important strength of our study was the inclusion of a large number of sipuleucel-T reports, thereby providing the first broad perspective of AEs in the postmarketing period, to our knowledge. However, our study also has limitations, including those inherent to passive surveillance reporting systems, such as voluntary (underreporting) and duplicate reporting (despite efforts to exclude duplicate reports), varying report quality, reporting bias, and absence of denominator data.^40,41^ In addition, data mining analyses do not implicate an association between reported AEs and use of sipuleucel-T but identify potential signals that may need further assessment.

## Conclusions

In summary, the present analysis—the largest postmarketing study to our knowledge to assess the safety profile of sipuleucel-T after its availability in the general population—did not raise new safety concerns. We found disproportionate reporting for labeled events, including infusion-associated reactions, infections, catheter-associated complications, and thromboembolic events, including TIA. Although the US FDA continues to monitor reports of AEs following sipuleucel-T administration, this overview of postapproval experience provides reassurance to health care professionals that the current USPI is consistent with the safety profile and serves as a resource for clinicians and patients as they consider the potential risks and benefits of treatment in the context of all available therapeutic options for CRPC.^42^

## References

[1] Dendreon Corporation. Provenge (sipuleucel-T): summary basis for regulatory action. http://wayback.archive-it.org/7993/20170723023808/https://www.fda.gov/downloads/BiologicsBloodVaccines/CellularGeneTherapyProducts/ApprovedProducts/UCM213114.pdf. Published April 29, 2010. Accessed June 17, 2019.

[2] U.S. Food and Drug Administration. Provenge (sipuleucel-T). https://www.fda.gov/vaccines-blood-biologics/cellular-gene-therapy-products/provenge-sipuleucel-T. Updated May 28, 2019. Accessed June 17, 2019.

[3] Dendreon Pharmaceuticals LLC. Provenge indication and important safety information. https://www.provengehcp.com/CancerTreatmentSideEffects/ImportantSafetyInformation. Accessed June 17, 2019.

[4] U.S. National Library of Medicine ClinicalTrials.gov. A registry of sipuleucel-T therapy in men with advanced prostate cancer (PROCEED). https://clinicaltrials.gov/ct2/show/study/NCT01306890. Updated June 7, 2019. Accessed February 12, 2019.

[5] Dendreon Pharmaceuticals LLC. Provenge (sipuleucel-T). https://www.provenge.com. Accessed May 30, 2019.

[6] Beer TM, Bernstein GT, Corman JM, Randomized trial of autologous cellular immunotherapy with sipuleucel-T in androgen-dependent prostate cancer. Clin Cancer Res. 2011;17(13):-. doi:10.1158/1078-0432.CCR-10-3223 21558406

[7] Ng L, Heck W, Lavsa S, The lack of predictors for rapid progression in prostate cancer patients receiving sipuleucel-T. Cancers (Basel). 2013;5(2):511-518. doi:10.3390/cancers5020511 24216988 PMC3730335

[8] Crawford ED, Higano CS, Shore ND, Hussain M, Petrylak DP Treating patients with metastatic castration resistant prostate cancer: a comprehensive review of available therapies. J Urol. 2015;194(6):1537-1547. doi:10.1016/j.juro.2015.06.106 26196735

[9] Lalani AA, Bossé D, McGregor BA, Choueiri TK Immunotherapy in the elderly. Eur Urol Focus. 2017;3(4-5):403-412. doi:10.1016/j.euf.2017.11.008 29183736

[10] Maia MC, Hansen AR A comprehensive review of immunotherapies in prostate cancer. Crit Rev Oncol Hematol. 2017;113:292-303. doi:10.1016/j.critrevonc.2017.02.026 28427519

[11] Gulley JL, Mulders P, Albers P, Perspectives on sipuleucel-T: its role in the prostate cancer treatment paradigm. Oncoimmunology. 2015;5(4):e1107698. doi:10.1080/2162402X.2015.1107698 27141392 PMC4839373

[12] Higano CS, Schellhammer PF, Small EJ, Integrated data from 2 randomized, double-blind, placebo-controlled, phase 3 trials of active cellular immunotherapy with sipuleucel-T in advanced prostate cancer. Cancer. 2009;115(16):3670-3679. doi:10.1002/cncr.24429 19536890

[13] Kantoff PW, Higano CS, Shore ND, ; IMPACT Study Investigators Sipuleucel-T immunotherapy for castration-resistant prostate cancer. N Engl J Med. 2010;363(5):411-422. doi:10.1056/NEJMoa1001294 20818862

[14] Small EJ, Schellhammer PF, Higano CS, Placebo-controlled phase III trial of immunologic therapy with sipuleucel-T (APC8015) in patients with metastatic, asymptomatic hormone refractory prostate cancer. J Clin Oncol. 2006;24(19):3089-3094. doi:10.1200/JCO.2005.04.5252 16809734

[15] US Food and Drug Administration. Questions and answers on FDA’s Adverse Event Reporting System. https://www.fda.gov/drugs/surveillance/fda-adverse-event-reporting-system-faers. Updated June 4, 2018. Accessed June 17, 2019.

[16] MedDRA. Introductory Guide: MedDRA version 20.1. https://www.meddra.org/sites/default/files/guidance/file/intguide_20_1_english_0.pdf. Published September 2017. Accessed May 21, 2019.

[17] ICH. Harmonisation for better health: MedDRA work products. http://www.ich.org/products/meddra.html. Accessed June 17, 2019.

[18] US Government Publishing Office. Postmarketing reporting of adverse experiences. 21 CFR section 600.80. https://www.govinfo.gov/app/details/CFR-2018-title21-vol7/CFR-2018-title21-vol7-sec600-80. Published April 1, 2018. Accessed July 2, 2019.

[19] Office of the Federal Register and the Government Publishing Office Electronic Code of Federal Regulations. https://www.ecfr.gov/cgi-bin/retrieveECFR?gp=&SID=83cd09e1c0f5c6937cd9d7513160fc3f&pitd=20180719&n=pt45.1.46&r=PART&ty=HTML#se45.1.46_1104. Accessed July 8, 2019.

[20] DuMouchel W Bayesian data mining in large frequency tables, with an application to the FDA spontaneous reporting system. Am Stat. 1999;53(3):177-190. doi:10.2307/2686093

[21] Szarfman A, Machado SG, O’Neill RT Use of screening algorithms and computer systems to efficiently signal higher-than-expected combinations of drugs and events in the US FDA’s spontaneous reports database. Drug Saf. 2002;25(6):381-392. doi:10.2165/00002018-200225060-00001 12071774

[22] Szarfman A, Tonning JM, Doraiswamy PM Pharmacovigilance in the 21st century: new systematic tools for an old problem. Pharmacotherapy. 2004;24(9):1099-1104. doi:10.1592/phco.24.13.1099.38090 15460169

[23] Ball R Methods of ensuring vaccine safety. Expert Rev Vaccines. 2002;1(2):161-168. doi:10.1586/14760584.1.2.161 12901555

[24] US National Library of Medicine. Dailymed. https://dailymed.nlm.nih.gov/dailymed/. Accessed June 17, 2019.

[25] Levy MM, Fink MP, Marshall JC, ; International Sepsis Definitions Conference 2001 SCCM/ESICM/ACCP/ATS/SIS International Sepsis Definitions Conference. Intensive Care Med. 2003;29(4):530-538. doi:10.1007/s00134-003-1662-x 12664219

[26] Bone RC, Balk RA, Cerra FB, ; ACCP/SCCM Consensus Conference Committee; American College of Chest Physicians/Society of Critical Care Medicine Definitions for sepsis and organ failure and guidelines for the use of innovative therapies in sepsis. Chest. 1992;101(6):1644-1655. doi:10.1378/chest.101.6.1644 1303622

[27] Churpek MM, Zadravecz FJ, Winslow C, Howell MD, Edelson DP Incidence and prognostic value of the systemic inflammatory response syndrome and organ dysfunctions in ward patients. Am J Respir Crit Care Med. 2015;192(8):958-964. doi:10.1164/rccm.201502-0275OC 26158402 PMC4642209

[28] Kaukonen KM, Bailey M, Pilcher D, Cooper DJ, Bellomo R Systemic inflammatory response syndrome criteria in defining severe sepsis. N Engl J Med. 2015;372(17):1629-1638. doi:10.1056/NEJMoa1415236 25776936

[29] Seymour CW, Liu VX, Iwashyna TJ, Assessment of clinical criteria for sepsis: for the Third International Consensus definitions for sepsis and septic shock (Sepsis-3). JAMA. 2016;315(8):762-774. doi:10.1001/jama.2016.0288 26903335 PMC5433435

[30] Flanigan RC, Polcari AJ, Shore ND, An analysis of leukapheresis and central venous catheter use in the randomized, placebo controlled, phase 3 IMPACT trial of sipuleucel-T for metastatic castrate resistant prostate cancer. J Urol. 2013;189(2):521-526. doi:10.1016/j.juro.2012.09.029 23253957

[31] Maki DG, Kluger DM, Crnich CJ The risk of bloodstream infection in adults with different intravascular devices: a systematic review of 200 published prospective studies. Mayo Clin Proc. 2006;81(9):1159-1171. doi:10.4065/81.9.1159 16970212

[32] Nolan ME, Yadav H, Cawcutt KA, Cartin-Ceba R Complication rates among peripherally inserted central venous catheters and centrally inserted central catheters in the medical intensive care unit. J Crit Care. 2016;31(1):238-242. doi:10.1016/j.jcrc.2015.09.024 26519981

[33] Bosco C, Bosnyak Z, Malmberg A, Adolfsson J, Keating NL, Van Hemelrijck M Quantifying observational evidence for risk of fatal and nonfatal cardiovascular disease following androgen deprivation therapy for prostate cancer: a meta-analysis. Eur Urol. 2015;68(3):386-396. doi:10.1016/j.eururo.2014.11.039 25484142

[34] Ehdaie B, Atoria CL, Gupta A, Androgen deprivation and thromboembolic events in men with prostate cancer. Cancer. 2012;118(13):3397-3406. doi:10.1002/cncr.26623 22072494 PMC3678973

[35] Van Hemelrijck M, Garmo H, Holmberg L, Absolute and relative risk of cardiovascular disease in men with prostate cancer: results from the Population-Based PCBaSe Sweden. J Clin Oncol. 2010;28(21):3448-3456. doi:10.1200/JCO.2010.29.1567 20567006

[36] Chen RT, Rastogi SC, Mullen JR, The Vaccine Adverse Event Reporting System (VAERS). Vaccine. 1994;12(6):542-550. doi:10.1016/0264-410X(94)90315-8 8036829

[37] Pérez-Vilar S, Díez-Domingo J, Gomar-Fayos J, Pastor-Villalba E, Sastre-Cantón M, Puig-Barberà J Post-licensure passive safety surveillance of rotavirus vaccines: reporting sensitivity for intussusception [in Spanish]. An Pediatr (Barc). 2014;81(2):77-85. doi:10.1016/j.anpedi.2013.10.027 24252603

[38] Rosenthal S, Chen R The reporting sensitivities of two passive surveillance systems for vaccine adverse events. Am J Public Health. 1995;85(12):1706-1709. doi:10.2105/AJPH.85.12.1706 7503351 PMC1615747

[39] Caram MEV, Ross R, Lin P, Mukherjee B Factors associated with use of sipuleucel-T to treat patients with advanced prostate cancer. JAMA Netw Open. 2019;2(4):e192589. doi:10.1001/jamanetworkopen.2019.2589 31002323 PMC6481456

[40] Alatawi YM, Hansen RA Empirical estimation of under-reporting in the U.S. Food and Drug Administration Adverse Event Reporting System (FAERS). Expert Opin Drug Saf. 2017;16(7):761-767. doi:10.1080/14740338.2017.1323867 28447485

[41] Alvarez-Requejo A, Carvajal A, Bégaud B, Moride Y, Vega T, Arias LH Under-reporting of adverse drug reactions: estimate based on a spontaneous reporting scheme and a sentinel system. Eur J Clin Pharmacol. 1998;54(6):483-488. doi:10.1007/s002280050498 9776440

[42] Ingrosso G, Detti B, Scartoni D, Current therapeutic options in metastatic castration-resistant prostate cancer. Semin Oncol. 2018;45(5-6):303-315. doi:10.1053/j.seminoncol.2018.10.001 30446166

